# The dynamic functional core network of the human brain at rest

**DOI:** 10.1038/s41598-017-03420-6

**Published:** 2017-06-07

**Authors:** A. Kabbara, W. EL Falou, M. Khalil, F. Wendling, M. Hassan

**Affiliations:** 1 INSERM, U1099, F-35000 Rennes, France; 2 University of Rennes 1, LTSI, F-35000 Rennes, France; 3 Azm Center for Research in Biotechnology and its Application, EDST, Lebanese University, Beirut, Lebanon; 4 CRSI research center, Faculty of Engineering, Lebanese University, Beirut, Lebanon

## Abstract

The human brain is an inherently complex and dynamic system. Even at rest, functional brain networks dynamically reconfigure in a well-organized way to warrant an efficient communication between brain regions. However, a precise characterization of this reconfiguration at very fast time-scale (hundreds of millisecond) during rest remains elusive. In this study, we used dense electroencephalography data recorded during task-free paradigm to track the fast temporal dynamics of spontaneous brain networks. Results obtained from network-based analysis methods revealed the existence of a functional dynamic core network formed of a set of key brain regions that ensure segregation and integration functions. Brain regions within this functional core share high betweenness centrality, strength and vulnerability (high impact on the network global efficiency) and low clustering coefficient. These regions are mainly located in the cingulate and the medial frontal cortex. In particular, most of the identified hubs were found to belong to the Default Mode Network. Results also revealed that the same central regions may dynamically alternate and play the role of either provincial (local) or connector (global) hubs.

## Introduction

The human brain is a complex network. Even at rest, spatially distributed brain regions are functionally connected, in a very organized way, to continuously share information with each other^1–4^. The intrinsic connectivity networks (ICNs), also known as Resting State Networks (RSNs), are now widely recognized and found to be quite consistent across subjects^1, 5–13^ as well as neuroimaging techniques^14–17^. The most commonly known RSNs are the default mode network (DMN), the dorsal attention network (DAN), the ventral attention network (VAN), the motor network, the visual network (VIS), and the auditory network (AUD).

Several studies have reported the existence of few critical regions that may play a key role in establishing and maintaining an efficient brain communication at rest. The presence of central brain regions or ‘hubs’ at rest has been revealed for structural^18–27^ and functional^28–32^ connections. In addition to their highly central role, recent studies have shown that these brain hubs tend to be densely interconnected with each other more than expected by chance, forming the so called rich-club organization of the human brain^33–35^.

Using Magnetoencephalography (MEG) or/and Electroencephalography (EEG), it was shown that these RSNs have an electrophysiological basis^16, 36–41^. In a preliminary study^32^, we used dense-EEG recordings to confirm the existence of brain regions playing the role of hubs in a static scenario. Yet, the main gain of using M/EEG is the excellent temporal resolution that allows the tracking of the temporal dynamics of RSNs at sub-second time scale, not reachable when using fMRI. Various MEG studies showed the crucial role of the DMN, and the cingulate cortex in particular, in maintaining efficient temporal communication in the whole brain^16, 42^. Other studies focused on assessing the temporal transitions between RSNs^40^. However, none of them looked at the temporal transition between brain regions, networks and modules over hundreds of millisecond time scale.

To tackle this issue, we collected dense-EEG data from 20 subjects sitting without performing any particular task. We then reconstructed the functional networks using EEG source connectivity approach as described in previous work^32, 43, 44^. Topologies of the identified networks were characterized in terms of node’s strength, vulnerability, betweenness centrality and clustering coefficient. In the present study, we extend our previous static analysis^32^ toward the study of the dynamic interactions between resting state networks. We have also explored the dynamic modularity and classified brain regions into provincial (intra-community) and connector (inter-community) hubs. Our results revealed the existence of a dynamic core network located mainly in the cingulate and the medial frontal cortex. We found that a large proportion of the brain hubs belong to the DMN. Results also revealed that the same brain hubs might dynamically change their actions and play the role of either provincial (segregation) or connector (integration) hubs.

## Results

The functional networks were estimated using dense EEG source connectivity method. As recommended in Hassan *et al*.^44^, we combined the weighted Minimum Norm Estimate (wMNE) and the Phase Locking Value (PLV) to reconstruct the dynamic of the cortical sources and compute the functional connectivity between these sources. This produced a fully connected, undirected and weighted networks (see Materials and Methods for details about the construction of the functional networks). In order to explore the advantages of the dynamic analysis, we performed our study in two ways: ‘static’ and ‘dynamic’. For the static approach, the functional connectivity was computed over the entire noise-free epoch duration (40 seconds). To examine the dynamics of the RSNs, we used a sliding window of 300 milliseconds in which PLV was calculated over its data points (see Materials and Methods for more details). This value was chosen as it represents the minimal time window size required to adequately compute PLV as recommended by Lachaux *et al*.^45^. Other time window sizes (1 s, 2 s, 10 s and 40 s) were also explored and results are reported in Figures S2 and S4 in the Supplementary Materials.

**Figure 1:**
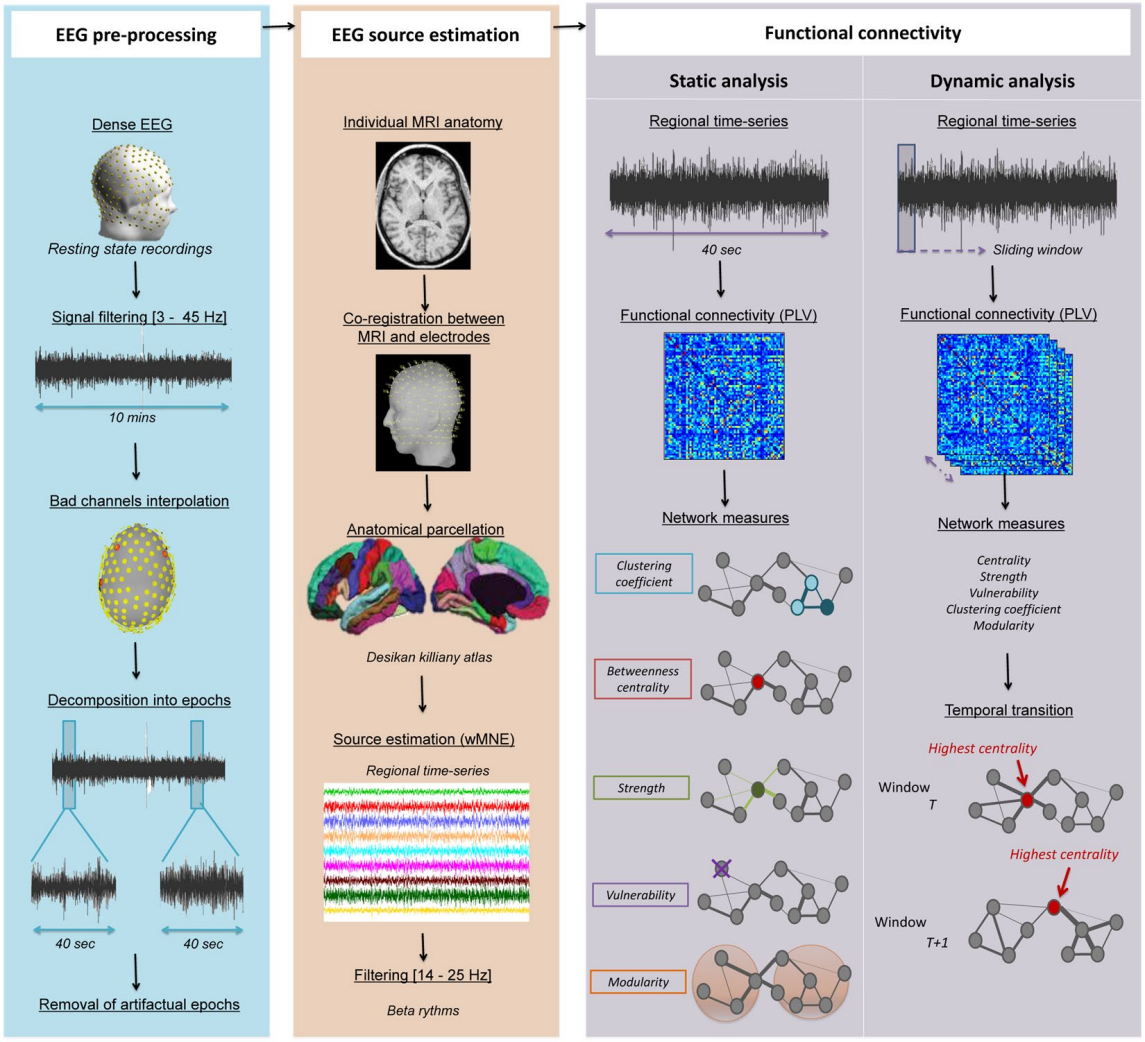
Structure of the investigation. *left*: Pre-processing of the dense-EEG data by interpolating channels and removing artifactual epochs, *middle*: Estimation of the EEG cortical sources using the weighted norm estimation method (wMNE). This step was followed by a projection of the source signals on the Desikan-killiany atlas, *right*: Quantification of the functional connectivity between the regional time series using the phase locking value (PLV). Two analyses were performed: (i) the static analysis in which PLV was computed over a segment of 40 s and (ii) the dynamic analysis in which PLV was computed over a 300 ms sliding window. The networks were then characterized by different graph measures (centrality, strength, vulnerability, clustering coefficient and modularity). The temporal transitions between networks/node’s characteristic across time were also performed.

We then identified the brain hubs by computing the centrality, vulnerability, strength and clustering coefficient measures of the different brain regions. These measures have been evaluated here since they represent the most commonly used metrics to detect brain hubs^4, 21, 22, 28, 31, 34, 42, 46–54^. In this context, Sporns *et al*.^54^ showed that a node can be defined as hub if it has an unusually high strength (a large number of connections) and centrality (the node lies on a high number of shortest paths) and a low clustering coefficient (the neighbours of a hub are not directly connected with each other). We also speculate, based on previous studies^19, 33, 48, 55, 56^, that adding the vulnerability metric to the spectrum of network measures can provide new insights into the definition of the hubness. Indeed, a node with high vulnerability is supposed to have a high influence on the global efficiency of the network^55^. In addition, we have classified hubs into provincials and connectors based on a combination between the participation coefficient and the within-degree module^24, 33, 54, 57–60^. The full pipeline of our study is summarized in Fig. 1.

### Static analysis

In this analysis, we computed the graph metrics (centrality, vulnerability, strength and clustering coefficient) using the entire signal length (40 s). We then quantified the difference between nodes distributions of each graph metric using a Wilcoxon test. Only nodes showing significant difference (*p* < 0.01, Bonferroni corrected) were retained, see Materials and Method section.

**Figure 2:**
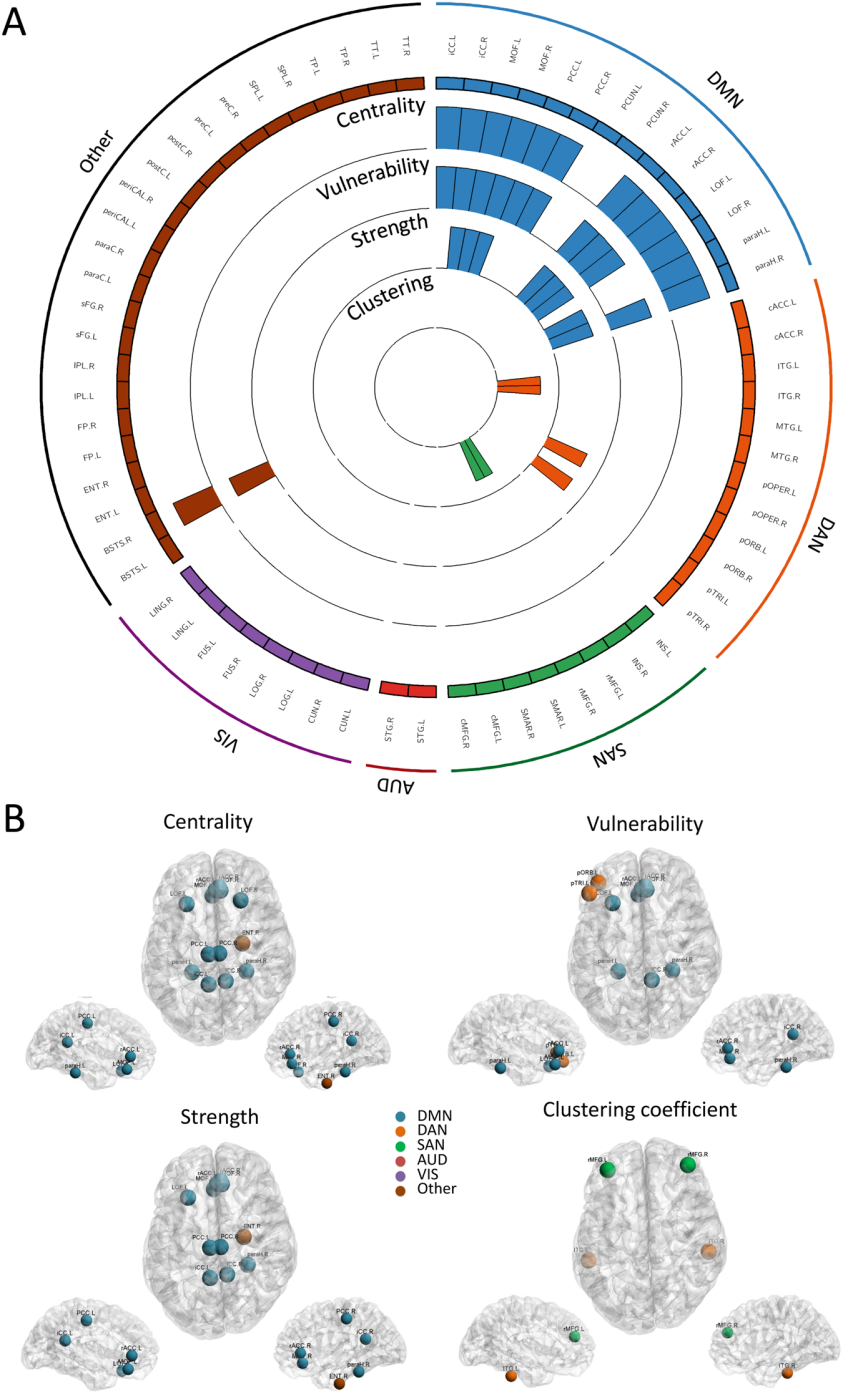
Static analysis: graph metrics. (**A**) The distribution of the four measures across the 68 brain regions. The circular barplots reflect from the outside inward: centrality, vulnerability, strength and clustering coefficient. The outermost ring shows the 68 brain regions obtained from the anatomical parcellation based on Desikan-Killiany atlas^61^, arranged by their assigned resting state networks: default mode network (DMN), dorsal attentional network (DAN), salience network (SAN), auditory network (AUD), visual network (VIS), see Table S1 in Supplementary Materials for more details about these assignments. We only showed the bars for significant nodes (*p* < 0.01, Bonferroni corrected). (**B**) The location of the significant brain regions on the cortical surface. The color of the node corresponds to which RSN is assigned. Names and abbreviations of the brain regions are listed in Table S1.

Figure 2A presents the results obtained for all subjects. It shows four circular barplots reflecting from the outside inward: centrality, vulnerability, strength and clustering coefficient. The outermost ring shows the 68 brain regions (obtained from the anatomical parcellation based on Desikan-Killiany atlas)^61^ arranged by their assigned resting state network (see Table 1 in Supplementary Materials). The figure also shows that the central nodes are L/R iCC, L/R PCC, L/R paraH, L/R MOF, L/R rACC, L/R LOF, L pTRI, L pORB and R ENT. The vulnerable nodes are R iCC, L/R paraH, L/R MOF, L/R rACC, L LOF, L pTRI, L pORB and R ENT. The L/R iCC, L/R PCC, R paraH, L/R MOF, L/R rACC, L LOF are the regions with highest strength while L/R ITG and L/R rMFG are the regions with highest clustering coefficients.

**Table 1:** A comparison between the identified brain hubs in our study with structural and functional previous studies.

Detected as hub in our study		Detected as hub in literature	
Region	Graph measure	Graph measure	Technique
iCC	Str, BC, Vuln: static, dynamic	Deg: ^18, 47^, Str: ^27^	DTI
	P, Z: static, dynamic	BC: ^27, 53^, PL: ^47^	fMRI
PCC	BC, Vuln: static	Deg: ^22, 27, 47^, Str: ^27, 29^	DTI
	P, Z: static, dynamic	BC: ^21, 22, 27, 31, 42^	fMRI
		RC: ^23^, PL: ^47^	MEG
rACC	Str: static, dynamic	Deg: ^22, 47^	DTI
	BC, Vuln: static	BC: ^22, 31, 49, 53^	fMRI
	P, Z: static, dynamic	RC: ^23, 24^, P, Z: ^27^	
MOF	Str, BC, Vuln: static, dynamic	Deg: ^22, 47^	DTI
	P, Z: static, dynamic	BC: ^21, 22, 31, 42, 49^	fMRI
		P, Z: ^27^, PL: ^47^	MEG
LOF	Str, Vuln: static, dynamic	Deg: ^22, 47^	DTI
	BC: static	BC: ^21, 22^	fMRI
	P, Z: static, dynamic		MEG
ParaH	Str, BC, Vuln: static, dynamic	BC: ^53^, RC: ^52^	DTI
	P, Z: static, dynamic	P, Z: ^53^	fMRI
FUS	Strength: dynamic	Deg: ^47^	fMRI
	P, Z: dynamic	BC: ^53^	
		PL: ^47^	
PCUN	P, Z: static, dynamic	Deg: ^18, 19, 22, 58^	DTI
		Str: ^29^, BC: ^18, 19, 22^	fMRI
		RC: ^23, 24, 52^, PL: ^47^	
		Vuln: ^18^	
		P, Z: ^27, 52, 58–60^	
LING	P, Z: dynamic	Deg: ^47^, PL: ^47^	fMRI
ParaC	P, Z: static	Deg, Str, BC: ^27^	DTI
		P, Z: ^27^	
Cunues	P, Z: dynamic	Deg: ^47^, PL: ^47^	DTI
		P, Z: ^24^, RC: ^24^	fMRI
periCal	P, Z: static	Deg, Str, BC: ^27^	DTI
		P, Z: ^27^	
ENT	Str: dynamic	×	×
	BC, Vuln: static		
pORB	Str: static, dynamic	×	×
	P, Z: static, dynamic		
pTRI	Str, Vuln: static, dynamic	×	×
	P, Z: static		

Abbreviations. Deg: degree, Str: strength, PL: path length, BC: betweenness centrality, P: participation coefficient, Z: within degree module and Vuln: vulnerability.

**Figure 3:**
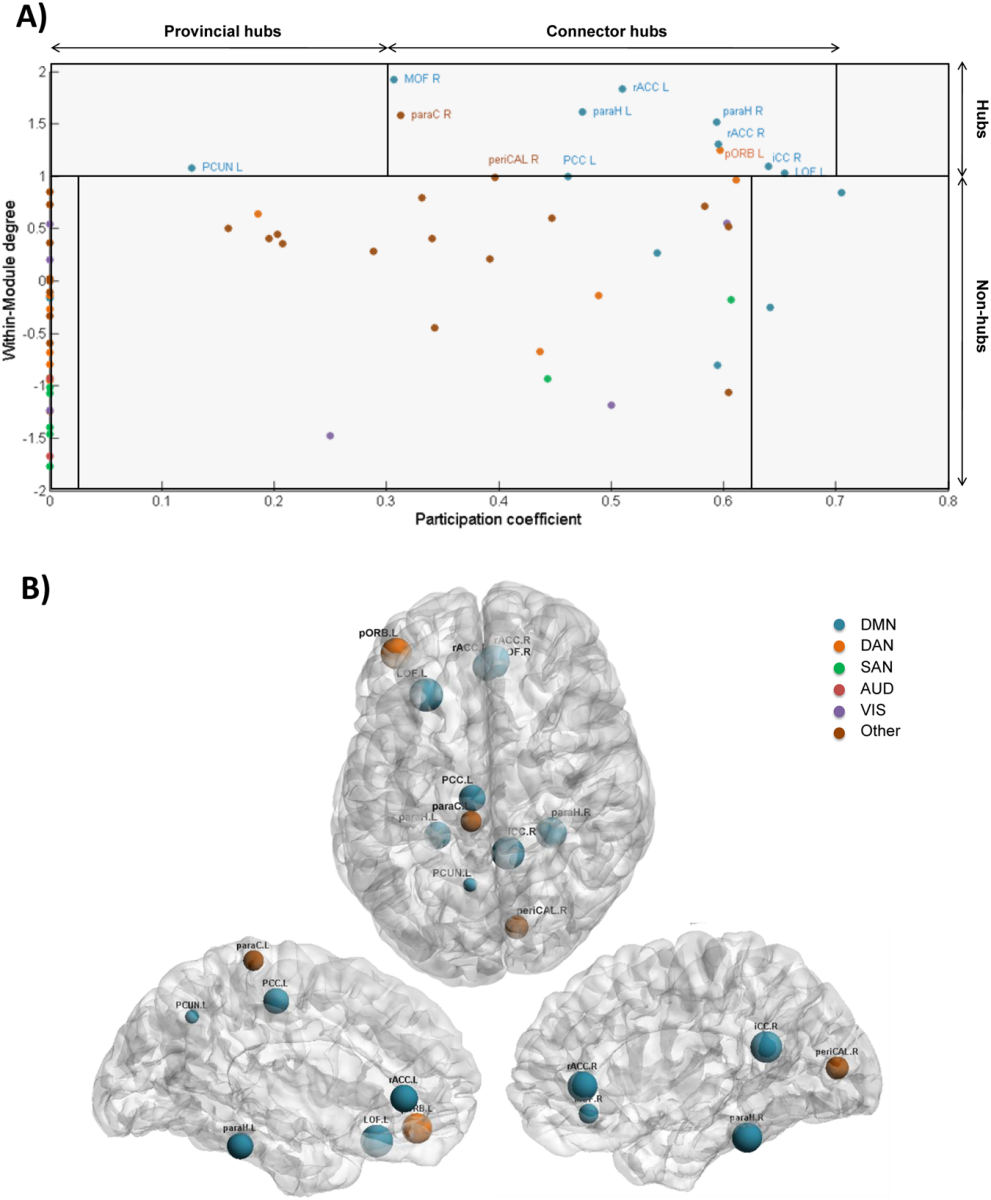
Static analysis: modularity. (**A**) The scatter plot of the participation coefficient and the within module degree for the 68 brain regions. Based on^57^, three main areas can be identified: Non-hubs, provincial and connector hubs. (**B**) The spatial locations of the identified hubs on the cortical surface. Names and abbreviations of the brain regions are listed in Table S1.

Results also demonstrated that a large proportion of the identified brain regions in terms of centrality (12/13), vulnerability (10/11) and strength (8/8) belong to the DMN (Fig. 2B). In contrast, one can notice that the nodes that have the highest clustering coefficients were distributed across the DAN and the SAN, while no significant node was belonging to the DMN. Brain regions were also classified into provincial hubs, connector hubs and non-hubs by computing the participation coefficient combined with the within-degree z-score of the association matrix obtained for all subjects (Fig. 3A), refer to Materials and Methods section for more details about the modularity analysis. Figure 3B illustrates the spatial locations of the resultant hubs on the cortical surface. We observe that a large number of hubs belong to the DMN (9/12) with the presence of one node belonging to the DAN and two nodes not belonging to any of the five analyzed RSNs. The PCUN region was depicted as a provincial hub, while L PCC, R MOF, L/R paraH, L/R rACC, R paraC, R periCal, R iCC, L pORB and L LOF regions are classified as connector hubs.

### Dynamic analysis

**Figure 4:**
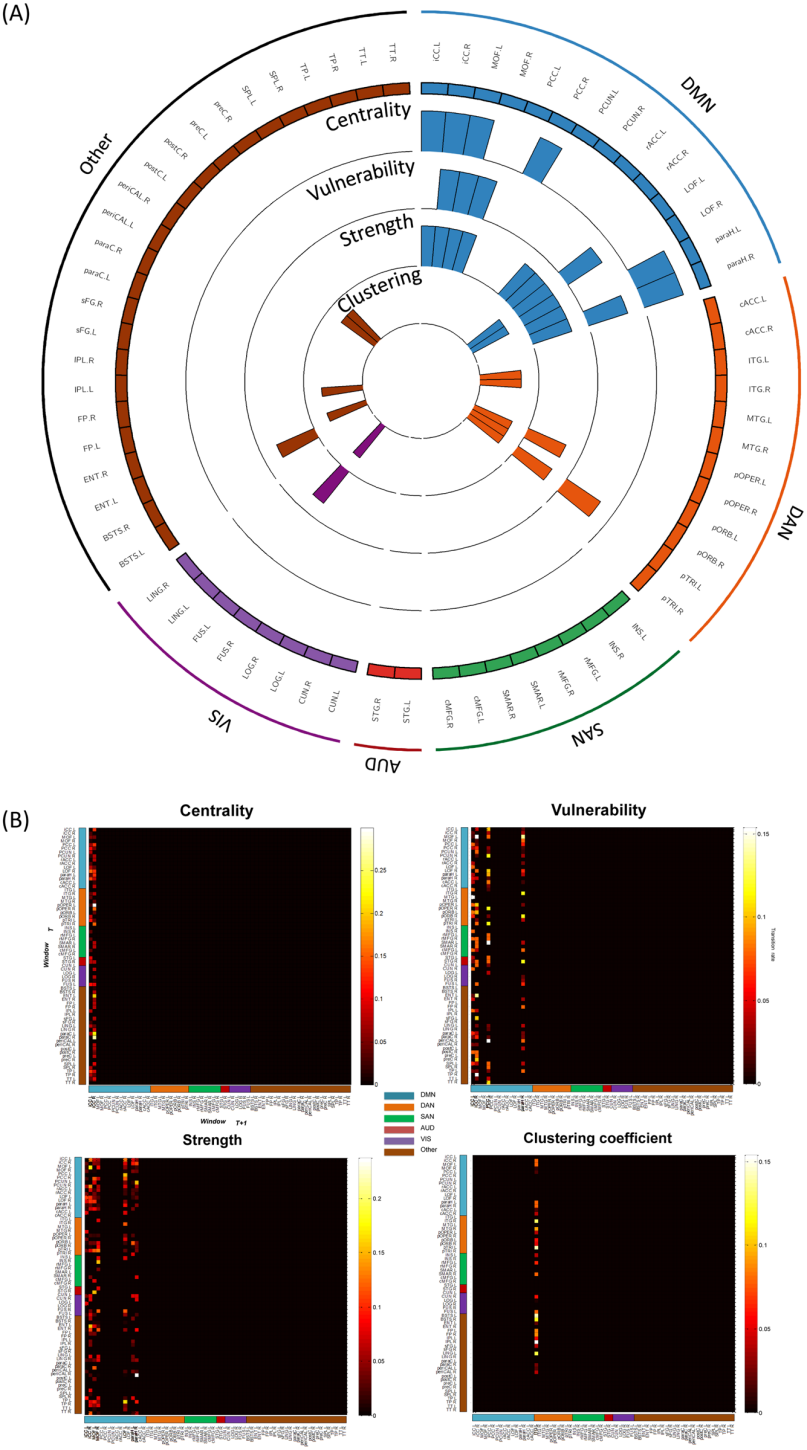
Dynamic analysis: graph metrics. (**A**) The distribution of the four measures across the 68 brain regions. The four circular barplots reflect (from the outside inward): centrality, vulnerability, strength and clustering coefficient. The outermost ring shows the 68 brain regions (obtained from the anatomical parcellation based on Desikan-Killiany atlas^61^), arranged by their assigned resting state networks: default mode network (DMN), dorsal attentional network (DAN), salience network (SAN), auditory network (AUD), visual network (VIS) (see Table S1 in Supplementary Materials). We only retain the bars for significant nodes (*p* < 0.01, Bonferroni corrected). (**B**) The temporal transitions between the 68 brain regions in terms of centrality, strength, vulnerability and clustering coefficient. Only significant columns are shown (*p* < 0.01, Bonferroni corrected). Names and abbreviations of the brain regions are listed in Table S1.

To investigate importance of the dynamic analysis, we applied the same above procedure for each sliding window. The centrality histogram depicts L/R iCC, R PCC, L MOF, and L/R paraH as significant regions. Concerning the vulnerability, the significant nodes are R iCC, R paraH, L/R MOF and L LOF. The nodes having the highest strength values are L/R iCC, L/R rACC, L/R paraH, L/R MOF, R ENT, L/R LOF and L FUS regions. Concerning the clustering parameter, L/R LOF, L/R ITG, L pTRI, L/R pORB, LFP, L/R postC, L FUS and L IPL regions showed the highest values (Fig. 4A). Very similar results were obtained using different time windows and thresholds (see Supplementary Materials, Figures S1 and S2).

We then investigated how the brain regions characteristics are fluctuating during time, and which regions are more frequently involved in the segregation/integration than others. To do that, we extracted at each time window the nodes with the highest centrality, vulnerability, strength and clustering coefficient values. We then computed the transition matrix which represents the number of changing times from one region to another for each of the graph metrics. The transition matrices illustrated in Fig. 4B demonstrate the significant (color-coded) columns (*p* < 0.01, Bonferroni corrected). One can state that the transition to nodes assigned to the DMN is very frequent compared to other RSNs. Importantly, results show that that there is a high probability of transition to L/R iCC according to centrality, vulnerability and strength. According to vulnerability, the columns corresponding also to L pCC and R paraH are significant. Furthermore, the transitions to L LOF, the L/R MOF, L/R paraH are higher in the strength transition matrix. However, there is no significant transition to any of the DMN nodes concerning the clustering coefficient, and the single remaining column corresponding to L ITG.

**Figure 5:**
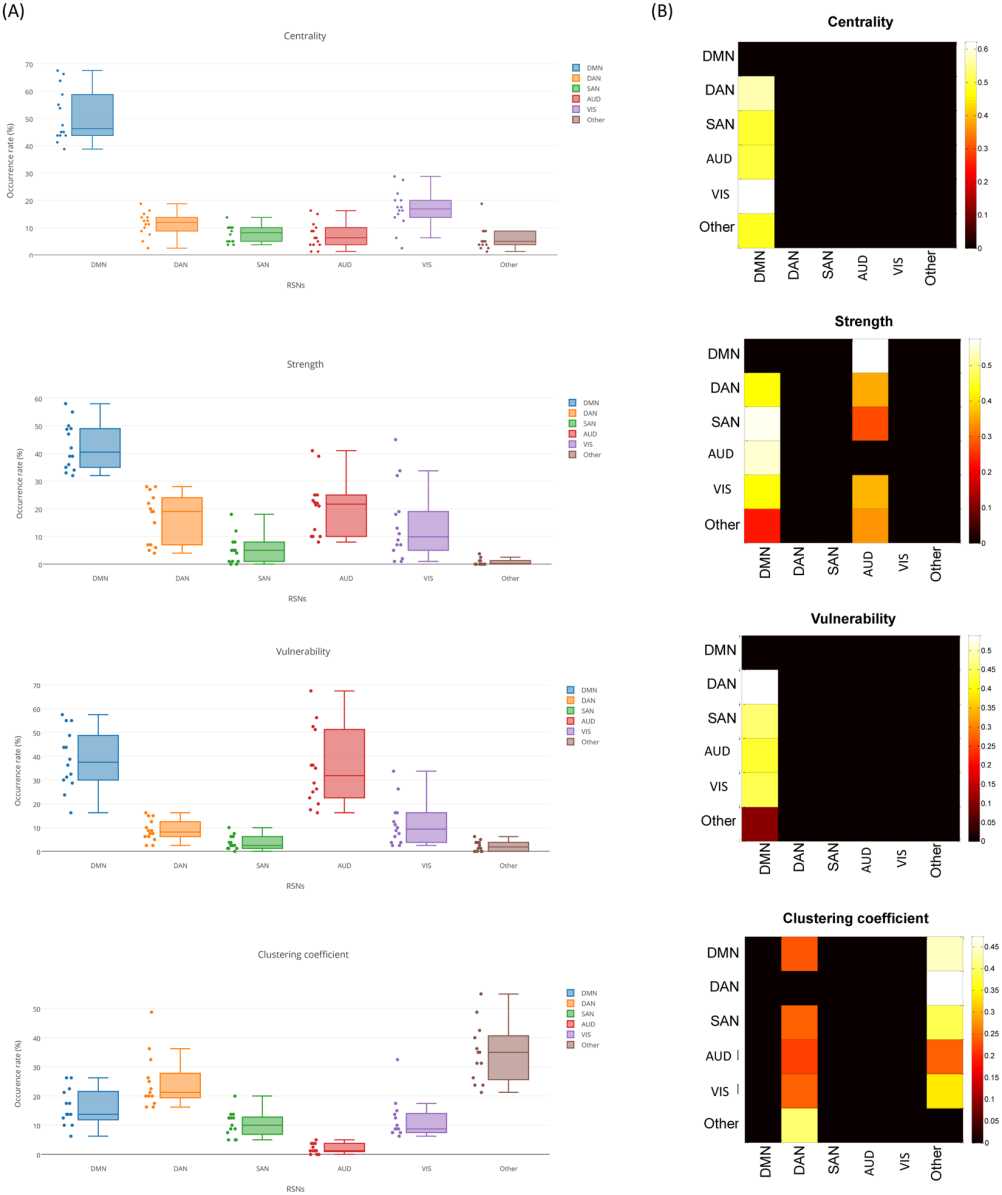
Dynamic analysis: networks transition. (**A**) The occurrence rates of the DMN, SAN, DAN, VIS, AUD and Other RSNs across time windows for all participants. (**B**) The temporal transitions between all networks across time windows for all participants. Only significant columns are shown (*p* < 0.01, Bonferroni corrected).

We have also evaluated the fractional occupancy of RSNs during time. Initially we extracted the significant nodes at each time window and then we associated the considered window to the RSN that contains the majority of these nodes (see Table S1 for nodes affiliations). After that, we computed the occurrence rates of each RSN across all segments and all participants for the four measures. The statistical test using Wilcoxon demonstrates a significant difference between DMN and other RSNs with regard to centrality and strength (*p* < 0.01). However, no significant difference was found between DMN and AUD according to the vulnerability measure. For the clustering coefficient, other RSNs occupancy rate is significantly different from that of DMN, DAN, SAN, AUD and VIS (Fig. 5A).

Interestingly, inspection of the transition matrices between RSNs reveals that there is a preference for the brain to move the centrality, vulnerability and strength characteristic to DMN rather than other networks (Fig. 5B). We also note that the vulnerability transition from the DMN to the AUD network is significantly considerable. Consistent with the previous results, the clustering coefficient transition to the DAN and ‘Other’ is the highest among the five known RSNs.

**Figure 6:**
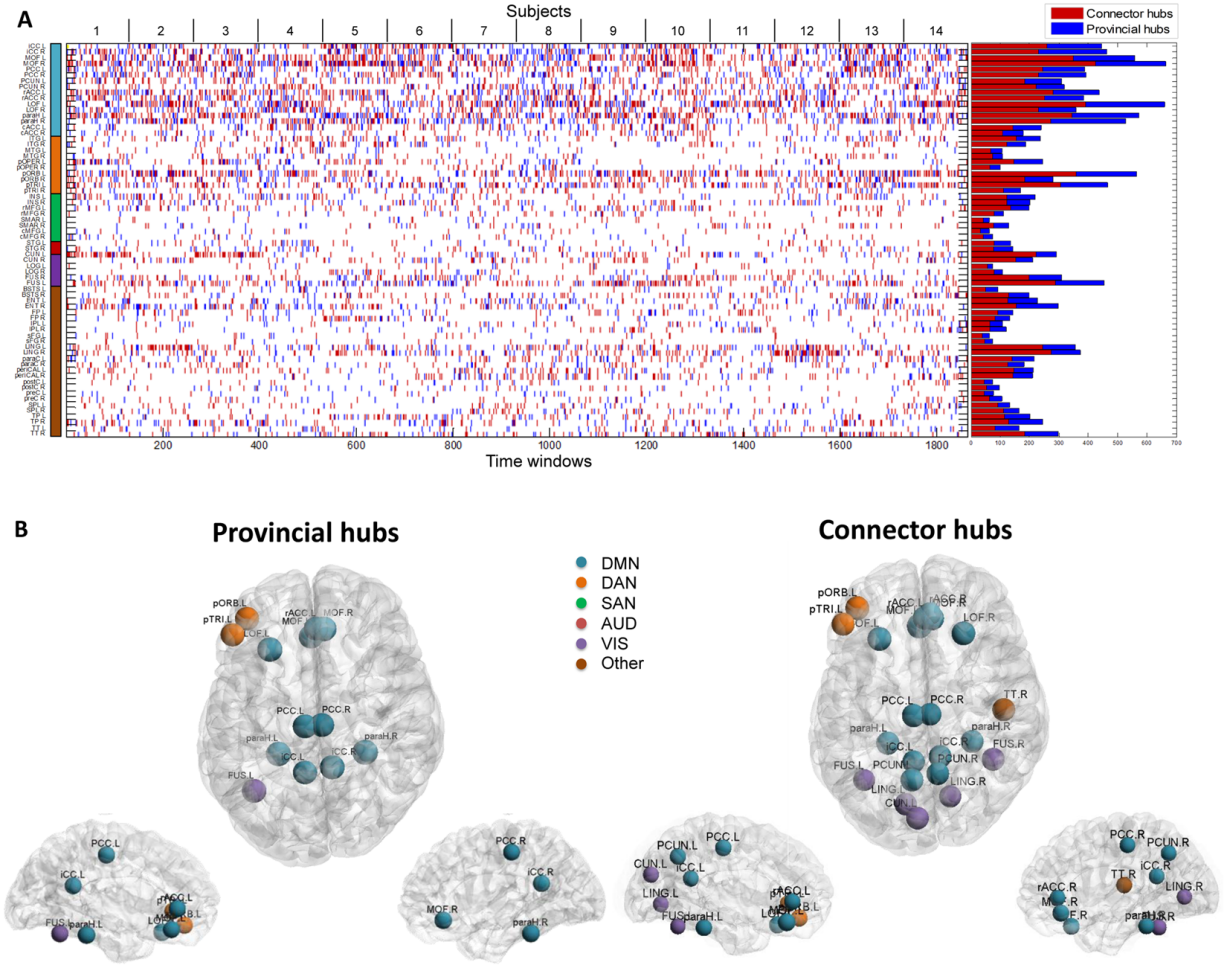
Dynamic analysis: modularity. (**A**) *left*: The variations of the node’s type (provincial vs. connector) across time for each of the 68 brain regions, *right*: The bar plots represent the number of times a node is considered as provincial hub (blue color) and as connector hub (red color). (**B**) The spatial distributions of significant provincial hubs, and significant connector hubs. Names and abbreviations of the brain regions are listed in Table S1.

Finally, we have explored how a brain region can change its functional role (segregation/integration) over time. Thus, we assigned each of the 68 brain regions to one of the three classes (non-hub, provincial hub, connector hub) at each time window. Figure 6A illustrates the results for all subjects. A selected row in the matrix presents the role variations of a specific brain region across time windows. A simple examination of this figure reveals that the same node can change its type (provincial/connector) from one time to another. To extract the brain regions that are significantly behaving as connector or/and provincial hubs, we performed a chi-squared test and retained the significant nodes (*p* < 0.01, Bonferroni corrected). Ten out of the thirteen significant provincial hubs were found to be in the DMN, two are in the DAN and one was assigned to the VIS network. We also found a large proportion of connector hubs included in the DMN with the presence of two nodes in the DAN, three nodes in the VIS and three nodes assigned to none of the five RSNs. A considerable observation here is that some nodes may change their function by dynamically alternating between provincial and connector hubs in the resting network across time. Among these nodes, we cite L/R iCC, L/R paraH, L FUS, L LOF, L/R MOF, L/R PCC, L pORB, and L pTRI. Results of significant provincial and connector nodes using different time windows are presented in Supplementary Materials, Figure S3.

## Discussion

There is growing evidence suggesting that the brain is a complex system of interacting functional units. This complex network was shown to be dynamic and network’s behaviour changes over time. In this context, the recent past years have seen a significant increase of interest for dense-EEG analysis of functional brain networks at the level of cortical sources. This approach, called EEG source connectivity, is conceptually very attractive as high spatiotemporal resolution networks can be directly identified in the cortical source space. This method was recently evaluated for its capacity to reveal relevant networks in the context of cognitive tasks^44^ and brain disorders^62^. It was then extended to track the spatiotemporal dynamics of functional brain networks^43^. More recently, we have performed a preliminary study using this technique combined with graph theory to explore the brain network architecture during rest in a static way^32^. However, the dynamic reconfiguration of resting brain network and its associated brain regions over short time scale (hundreds of millisecond) remains elusive. In this study, we used the dense-EEG data combined with graph theory analysis to characterize the fast dynamic reconfiguration of the brain networks at rest.

In this paper, we have investigated the dynamic behaviour of the functional brain networks during rest over a very short time scale (<second). This has never been done before. We have also quantified the modular architecture of the dynamic brain networks and have extracted the local (provincial) and global (integrator) brain regions that play a key role in maintaining the communication between brain regions. We also showed that the same regions can play the same role (provincial or integrator) during a given time period. Again, these methodological aspects and the results are novel.

The main originality of this work is the combination of source connectivity analysis with graph theoretical study to explore the dynamics of node’s characteristics (centrality, vulnerability, strength and clustering), networks and modules over hundreds of milliseconds time scale which cannot be reached when using fMRI. Interestingly, the source connectivity method is a recently developed method used to identify functional networks at the cortical level from scalp dense-EEG recordings.

Our results showed mainly that the dynamic analysis of the RSNs at few hundreds of milliseconds time scale revealed valuable characteristics of the brain regions centrality and ‘hubness’. We showed that the human brain holds a dynamic functional core network of a set of central brain regions that dynamically ensure both segregation and integration processes. By classifying the brain regions into local and global hubs using the participation coefficient and the within-module degree, we showed for the first time that same brain region can dynamically switch its function between provincial (segregation) and connector (integration) hubs. Results are further discussed hereafter.

### Network hubs in the brain

Identifying brain regions that have a strong influence on information segregation and integration in the brain network is a key issue to characterize the brain functions. To the best of our knowledge, this is the first attempt to identify functional hubs based on EEG source connectivity using graph theory approach. It is therefore essential to substantiate its usefulness by comparing the obtained results to prior studies. For this end, we have selected all the nodes detected here as hubs in terms of centrality, vulnerability, strength and/or modularity-based method, and compared them to those detected previously using other neuroimaging techniques (DTI, fMRI, and MEG). We found considerable overlapping between our results and previous results for most brain regions, while three brain regions were only detected as hubs in our study (Table 1). The Table 1 shows the brain regions identified as hubs in our study in both static and dynamic approach and the corresponding graph measures. The table shows also if these regions were identified as hubs previously using other neuroimaging techniques.

To identify brain hubs, many graph measures have been used. The simplest commonly used way is the detection of highest-degree nodes. This approach has been used by several studies^13, 30, 63, 64^. Others proposed to combine the degree and path length metrics^31, 47, 53, 65, 66^. Here, we evaluated the most commonly used graph measures in order to obtain a possible convergence between several measures. Interestingly, Table 1 shows that the iCC, paraH and MOF regions are detected in both static and dynamic analysis independently of the graph measure used.

We hypothesized that a consistent hub node has a high centrality (the node lies a high number of shortest paths), high strength (the hub has a large number of connections), high vulnerability (the removal of the hub has a dramatic effect on the efficiency of the network) and low clustering coefficient (the neighbors of a hub are not directly connected with each other). Based on this definition of “hubness”, the R iCC, L/R MOF and R paraH regions are shown to be the strongest hubs as demonstrated in our static and dynamic analysis.

Several previous studies have also combined the participation coefficient and the within module degree to identify brain hubs^24, 33, 54, 58–60^. This method also allows the classification of hubs into two categories: provincial and connector. Using this approach, most of the hubs obtained in our study were intersecting with the already defined hubs using centrality, vulnerability and strength. Among these nodes, we list the L/R iCC, L/R paraH, L/R MOF, L/R PCC, L/R rACC, L/R pORB, L/R LOF, L/R FUS. Moreover, combining empirical results from structural and functional studies demonstrated a strong agreement between these hubs and the previously defined connector/provincial hubs. The PeriCal has also been detected as a provincial hub in Hagmann *et al*.^27^. The paraH was shown to play the role of connector hub in He *et al*.^53^. Similarly, the rACC region was identified as a connector in various studies^27, 53^ and paraC was detected as a connector as reported in Hagmann *et al*.^27^. Additionally, while the PCUN was considered as a provincial hub in some studies^58, 60^, it was identified as a connector hub in others^27, 34^. The MOF was identified as provincial in Hagmann *et al*.^27^ and as connector hub in Meunier *et al*.^59^.

### Hubs and RSNs

There is a current debate whether the brain hubs are included in a single functional network, or are distributed among multiple RSNs serving as inter-links between these functional networks. While many studies support the first hypothesis^16, 41, 58^, others suggest that hubs form an infrastructure for communication between RSNs^24, 33^. Our results show that the brain hubs are distributed among the DMN (the cingulate, parahippocampal and prefrontal cortex regions), the DAN (parsorbitalis, and parstriangularis regions) and the VIS (cunues, lingual and fusiform). However, one can notice that the DMN regions provide the largest contribution to the network segregation/integration. This is revealed by a high fractional occupancy associated to the DMN compared to other RSNs, suggesting a frequent transition to this network in the centrality, vulnerability and strength temporal transition analysis. Having the majority of hubs included in the DMN corroborates with the fact that DMN is the most dominating RSN^1, 10^. Similar results were reported in the literature^10, 67, 68^, where an important overlap between hubs and the DMN was observed. Furthermore, a study that explored the rich club organization of the human brain showed that the rich club nodes cross-link with the majority of RSNs and that the largest proportion belong to the DMN^33^.

### Dynamic core network

Most studies in RSNs were performed in a static way i.e. networks were identified over the entire recording (called also ‘stationary’ analysis). The assumption that the connectivity between brain regions is static throughout the resting recording was criticized in several studies^38^. In particular, Allen *et al*.^39^ reported that the functional connectivity states derived at rest from the dynamic analysis strongly differ from the patterns obtained using the static approach. Accordingly, Calhoun *et al*.^69^ introduced the term “chronnectome” to describe that the patterns of coupling among brain regions are dynamic and consistent over time.

Performing the analysis over the entire segment (40 s window length in our analysis) offered a global view about the characteristics of the RSNs. However, the sole static analysis prevents the exploration of how the brain regions/networks are reconfiguring at sub-second time scale. Moreover, examining the transition between nodes in terms of graph metrics allowed us to investigate how brain hubs are alternating between each other with time. Importantly, a unique finding has also been offered by tracking the dynamics, is the study of the hub’s type variations over time. In fact, a hub node has been usually considered as either provincial or connector hub^23, 27, 34, 53, 58–60^. However, we revealed that the same brain region can play the role of provincial hub or connector hub at two different times for same subject at rest. These findings are expected since the same regions have been detected as provincial hubs in some previous studies, and as connector hubs in others. For example, the PCUN was found to be a provincial hub in refs 60, 58 and a connector hub in refs 27, 34. Similarly for the MOF that was identified as provincial in ref. 27 and as connector hub in ref. 59. A possible explanation of these results is that these hub regions may participate in both the local segregation of the information and the global integration over the whole network.

### Methodological considerations

In this study, we used a proportional threshold (highest 10% of the edge’s weights) to remove weak connections of the functional connectivity matrices. Garisson *et al*.^70^ showed that network measures are stable across proportional thresholds contrary to absolute thresholds. Nevertheless and in order to ensure that the obtained results are not sensitive to the threshold value, we performed our analysis across a range of proportional thresholds (ranging from 5 to 20%) and realized the stability of our results across thresholds (see Supplementary Materials Figure S1). Results showed slight differences between the several threshold values. However, the overall conclusion of the study remains intact.

The time window used in the dynamic analysis corresponds to the minimal length that can be used to adequately compute PLV, as recommended by Lachaux *et al*.^45^. In order to verify the reproducibility of the obtained results, we repeated our analysis while changing the size of the selected window (300 ms - 1 s - 2s-10s). A high degree of agreement among these analyses was found, see Supplementary Materials Figures S2, S3. One can notice that most brain hubs were always located in the DMN for all time window sizes.

Here, we presented the results obtained by performing the study on beta rhythms based on previous findings^16, 17, 71, 72^. To verify the importance of beta band, we performed the same analysis on the broad-band (3–45 Hz), theta (3–7 Hz), alpha (7–13 Hz) and beta (14–25 Hz) frequency bands. We have evaluated the influence of the frequency band on the DMN occurrence, see Supplementary Materials Figure S4. The statistical test using Wilcoxon shows a significant difference between the DMN occurrences in beta, compared to theta and alpha (p < 0.01). However, no significant difference was found between DMN’s occupancy in beta compared to the broad-band.

From a methodological point of view, several issues should be discussed when reconstructing the sources from scalp EEG signals. In fact, the number of source dipoles is much larger than the number of electrodes, making the inverse problem ill-posed. This required adding several physical and mathematical constraints to solve the inverse problem. In the case of choosing the wMNE as an inverse solution, the main assumption was to find a solution with lowest energy. This assumption is generally explained by the economic energy cost of the brain during information processing. However, compared to other inverse solutions, the wMNE implies relatively few hypotheses, (see review in Becker *et al*.^73^ for more detailed comparison between inverse solution’s assumptions). Moreover, the lead-field matrix is underdetermined, and an accurate description of the head model will positively affect the quality of solutions. Here, we reduced the effect of this problem by computing a realistic subject-specific head model using each individual anatomical MRI image. In addition, the networks identified using EEG source connectivity are limited to the cortex as the sub-cortical regions are not easily accessible from scalp EEG recordings.

As an emerging technique, the evaluation of EEG source connectivity method is crucial. The question is to determine to what extent the functional brain networks identified from EEG source connectivity correspond to those that are actually activated during considered brain processes (resting state, cognitive task). For this purpose, we used (i) real data recorded during a cognitive task^43, 44^ and (ii) simulated data using biophysical/physiological modelling and real epileptic data^62^. In Hassan *et al*.^44^, the method was used to estimate the networks involved during a picture naming task for which a solid background was available regarding activated brain regions and networks. In brief, we performed a comprehensive literature review on these networks mainly obtained from neuroimaging techniques such as fMRI, MEG, depth EEG and PET. From this review, a “reference” network could be determined. It was used as a ground truth to define a performance criterion about the accuracy of networks obtained from EEG source connectivity. Interestingly, we tested a large number of combinations between the inverse solution and functional connectivity measures. For one combination (wMNE/PLV), the estimated network, activating during the cognitive task (500–700 ms), was found to spatially match the reference network. The above described work was then extended from static to dynamic analysis during the same cognitive task. We showed that the EEG source connectivity method was able to track the spatiotemporal dynamics of activated brain networks from the onset (presentation of the visual stimuli) to the reaction time (articulation). Estimated dynamic networks were also found to match previously-reported regions/networks, as identified with other techniques such as depth-EEG and MEG. More recently, a study was performed in the context of epilepsy where a physiologically-plausible computational model of epileptogenic networks was used as a ground truth. Simulated scalp-EEG signals were used to evaluate the performance of EEG source connectivity methods in term of “re-estimating” reference large-scale networks modelled at neocortical level. Again, the combination that showed the highest similarity between reference and estimated networks was the wMNE/PLV, used in the present paper.

Regarding the resting state data analyzed in the current paper, the only ‘ground-truth’ that could be considered are the fMRI recordings. Although fMRI data was not available for the healthy volunteers of our study, we didn’t ignore this issue and we have compared our results with those reported in literature using fMRI and DTI (Table 1). Qualitative comparison showed strong matching between brain hubs identified from our EEG-based methods, on the one hand, and brain hubs reported elsewhere and based on fMRI/DTI, on the other hand.

Furthermore, we assumed that by taking into account the whole atlas regions without any prior selection of particular region may give more straightforward results. Here, we used 68 anatomical ROIs to define the nodes in the brain network. There is no clear consensus about how to select the appropriate number of nodes that represent the large-scale networks. On one hand, choosing finer segmentation may increase the spatial resolution. On the other hand, keeping a reduced number of ROIs may help removing the spurious links that occur between spatially adjacent sources. In this regard, we assume that 68 regions were sufficient to investigate the global characteristics of the resting state networks while minimizing the problem of spurious connections between ‘very close sources’. Although the functional connectivity at the source level reduces the effect of the field spread, they do not suppress its effects completely. In this context, few strategies have been proposed to remove zero-lag correlations before performing any connectivity analyses^72, 74^. Others suggest only keeping the long-range connections^16, 41, 42^. However, these methods suppress important correlations that may occur at zero-lag^37^ or even among close regions.

In our study, we evaluated the possible effects of the field spread on our results by assessing the relationships between the average Euclidian distance of brain regions and their centrality, strength, clustering coefficient, participation index, and the within-module degree values. For each measure, the Euclidian distance of a node was calculated by averaging the distance between the node and all other nodes that affect the measurements. Our results showed that a large proportion of nodes have long connections with high metrics values. Furthermore, there was neither significant correlation between the betweenness centrality of a node and its average distance (ρ = −0.0627, *p* > 0.05), neither for the clustering coefficient (ρ = −0.0374, *p* > 0.05), the participation coefficient (ρ = −0.076, *p* > 0.05) and the within degree module (ρ = 0.013, *p* > 0.05). The correlation between the strength and the distance was statistically significant with a positive correlation value (ρ = 0.5, *p* < 0.01). This implies that the used metrics were not affected by the spurious short connections and that a high number of long-range connections were presented.

## Materials and Methods

### Data acquisition and pre-processing

The full pipeline of our study is summarized in Fig. 1. Data were recorded from twenty participants. All experiments were performed in accordance with the relevant guidelines and regulations of the National Ethics Committee for the Protection of Persons (CPP), (*BrainGraph* study, agreement number 2014-A01461- 46, promoter: Rennes University Hospital), which approved all the experimental protocol and procedures. Written informed consents were obtained from all participants in the study.

Structural MRI and EEG dense recordings (256 channels, EGI, Electrical Geodesic Inc.) were collected for each subject. During the acquisition, the subjects were asked to relax for 10 minutes with their eyes closed without falling asleep. Electrodes impedances were kept below 10 kΩ. EEGs were sampled at 1000 Hz, band-pass filtered within 3–45 Hz, and segmented into non-overlapping 40 s long epochs. After visual inspection, the segments that have substantial noise not due to brain activity (amplitudes over ± 80 μV) have been marked and excluded from the analysis. For some subjects, few electrodes with poor signal quality have been identified and interpolated using the surrounding channels activities. The artifact-free segments (four segments per subject on average) were then used for source estimation. The preprocessing steps were performed using EEGLAB^75^ and Brainstorm^76^ open source toolboxes.

A realistic head model was constructed by segmenting the MRI using Freesurfer software package^77^. The individual MRI anatomy and EEGs data were co-registered through the identification of the same anatomical landmarks (left and right pre-auricular points and nasion). The lead field matrix was then computed for a cortical mesh with 15000 vertices using OpenMEEG^78^. The regional time series were filtered in the beta band [14–25 Hz], in which many previous studies have reported its importance in driving large-scale spontaneous neuronal interactions^16, 17, 71, 72^. An atlas-based approach was used to project EEG signals onto an anatomical framework consisting of 68 cortical regions identified by means of the Desikan-Killiany^61^ atlas using Freesurfer^77^, http://freesurfer.net/, see Table S1 for more details about the names and abbreviations of these regions.

### Brain networks construction

Functional networks were computed using a recently proposed approach called ‘dense-EEG source connectivity’^43, 44^. It included two main steps: (i) solving the EEG inverse problem to reconstruct the temporal dynamics of the cortical regions at source level and (ii) measuring the functional connectivity between these reconstructed regional time series.

#### Source estimation

According to the linear discrete equivalent dipole model, EEG signals X(t) recorded from Q channels (Q = 256 in our case) can be expressed as linear combination of P time-varying current dipole sources S(t):

1 $$X(t)=G.S(t)+N(t)\,$$

where G is the lead field matrix and N(t) is the additive noise. G was computed from a multiple layer head model (volume conduction) and from the position of the Q electrodes. Here we used the Boundary Element Method (BEM) as a numerical method to compute realistic head models. We computed the lead field matrix using OpenMEEG^78^. In addition, the noise covariance matrix was calculated over a long segment of the resting recordings.

After calculating G and N(t), the inverse problem consists of estimating the parameters of the dipolar sources $\hat{{\rm{S}}}$(t) (notably the position, orientation and magnitude). As this problem is ill-posed (P ≫ Q), physical and mathematical constraints have to be added to find a single solution among the many solutions that minimize the residual term in the fitting of dense EEG signals. Using segmented MRI data, the source distribution can be constrained to a field of current dipoles homogeneously dispersed over the cortex and normal to the cortical surface. Precisely, in the source model, the electrical contribution of each macro-column to scalp electrodes can be represented by a current dipole located at the center of gravity of each triangle of the 3D mesh and oriented normally to the triangle surface. Using this source space, the weighted Minimum Norm Estimate (wMNE) method only estimates the moment of dipole sources. The wMNE compensates for the tendency of classical MNE to favor weak and surface sources (Hämäläinen and Ilmoniemi 1994). This is done by introducing a weighting matrix W_X_:

2 $${\hat{{\rm{S}}}}_{{\rm{wMNE}}}={({{\rm{G}}}^{{\rm{T}}}{{\rm{W}}}_{{\rm{X}}}{\rm{G}}+\lambda {\rm{I}})}^{-1}{{\rm{G}}}^{{\rm{T}}}{{\rm{W}}}_{{\rm{X}}}{\rm{X}}$$

where matrix W_X_ adjusts the properties of the solution by reducing the bias inherent to MNE solutions. Classically, W_X_ is a diagonal matrix built from matrix G with non-zero terms inversely proportional to the norm of the lead field vectors. λ is a regularization parameter computed relatively to the signal to noise ratio (λ = 0.2 in our analysis).

#### Functional connectivity

We used the phase locking value metric to compute the functional connectivity between the 68 reconstructed regional time-series. The combination of wMNE/PLV was shown to be very efficient to precisely identify cortical brain networks from scalp EEG during cognitive activity^43, 44^. As described in Lachaux *et al*.^45^, the phase locking value between two signals *x* and *y* is defined as:

3 $$PLV(t)=|\frac{1}{\delta }{\int }_{t-\delta /2}^{t+\delta /2}exp(j({\phi }_{y}(t)-{\phi }_{x}(t))d\tau |$$

where ${\phi }_{y}(t)$ and ${\phi }_{x}(t)$ are the unwrapped phases of the signals *x* and *y* at time *t*. The Hilbert transform was used to extract the instantaneous phase of each signal. *δ* denotes the size of the window in which PLV is calculated.

To explore the advantage of the dynamic analysis, we performed our study in both ‘static’ and ‘dynamic’ ways. For the static way, the functional connectivity was computed over the entire noise-free epoch duration (40 seconds). To examine the dynamics of the RSNs, we used a sliding window in which PLV was calculated over its data points. As recommended by Lachaux *et al*.^45^, the window length should be larger than $\frac{6}{central\,frequency}$ where 6 is the number of ‘cycles’ at the given frequency band. Having a central frequency of 19.5 Hz for the beta band, the smallest window length can be used is 300 milliseconds. Other frequency bands and other time window size will also described in the study.

### Network analysis

Networks can be illustrated by graphs, which are sets of nodes (brain regions) and of edges (connectivity values) between those nodes. We constructed graphs of 68 nodes (i.e. the 68 previously identified cortical regions) and used all information from the functional connectivity (phase locking value) matrix^79, 80^. This gave fully connected, weighted and undirected networks, in which the connection strength between each pair of vertices (i.e. the weight) was defined as their connectivity value.

We quantified the network’s nodes using several graph metrics:

#### Betweenness Centrality

The importance of a node is proportional to the number of paths in which it participates^51^. Thus, a way to find the critical nodes is to calculate the betweenness centrality of each node:

4 $$B{C}_{i}=\sum _{i,j}\frac{\sigma (i,u,j)}{\sigma (i,j)}$$

where $\sigma (i,u,j)$ is the number of shortest paths between nodes *i* and *j* that pass through node *u*, $\sigma (i,j)$ is the total number of shortest paths between *i* and *j*, and the sum is over all pairs *i*, *j* of distinct nodes.

#### Vulnerability

The vulnerability of a specific node can be defined as the reduction in performance when the node and all its edges are removed:

5 $${V}_{i}=\frac{E-{E}_{i}}{E}$$

where *E* is the global efficiency of the network before any attach, and ${E}_{i}$ is the global efficiency of the network after attacking the node *i* ^50^.

#### Strength

The strength of a node is the sum of the weights of its corresponding edges:

6 $${S}_{i}=\sum _{j}{w}_{ij}$$

where ${w}_{ij}$ is the weight of the edge linking the node *i* to the node *j* ^81^.

#### Clustering coefficient

The clustering coefficient of a node in a graph quantifies how close its neighbors are to being a clique^82^.

The network measures and visualization were performed using BCT^83^ and BrainNet viewer^84^, respectively. The above-mentioned network measures were normalized, that is, they were expressed as a function of measures computed from random networks. We generated 500 surrogate random networks derived from the original networks by randomly reshuffling the edge weights. The normalized values were computed by dividing the original values by the average of the values computed on the randomized graphs.

#### Modularity

Several algorithms have been proposed to decompose a network into modules or communities of high intrinsic connectivity and low extrinsic connectivity (Simon 1962). Due to the so-called degeneracy problem^85^, the modules of a same network differ from a run to another and from a module detection algorithm to another. With the aim to assess the consistency of modules affiliation, we applied the consensus clustering process as follows:

- Generate a set of partitions of the same network using three community detection methods 100 times (Newman algorithm^86^, Louvain algorithm^87^ and Infomap algorithm^88^).
- Compute the association matrix for all possible partitions^89–91^. This step results in a 68*68 matrix where the element ${A}_{i,j}$ represents the number of times the nodes *i* and *j* are assigned to the same module across the runs and algorithms.
- Compare the consensus matrix to a null model association matrix generated from a permutation of the original partitions^92^ and keeping its significant values^92^.
- Re-cluster the resultant association matrix using Louvain method.

Once a network has been partitioned, we classify the 68 nodes into three main categories (non hubs, provincial and connector hubs) by considering the variations of two measures used to quantify nodes connectivity within and between modules. The first one is the within-module degree z-score that express the number of links a node makes to other nodes in the same module:

7 $${Z}_{i}=\frac{{K}_{i}({m}_{i})-\overline{K({m}_{i})}}{{\sigma }_{k({m}_{i})}}$$

where ${K}_{i}({m}_{i})$ is the within-module degree of the node *i*, $\overline{K({m}_{i})}$ is the mean of within module degree of nodes assigned to the same community as node *i*, and ${\sigma }_{k({m}_{i})}$ is the standard deviation. Positive z-scores indicate that a node is highly connected to other members of the same community; negative z-scores indicate the opposite. In our study, nodes with ${Z}_{i} > 1.5\,\,$were considered as hubs, and nodes with ${Z}_{i} < 1.5$ were considered as non-hubs.

We then focused on discriminating provincial and connector hubs based on a second metric known as participation coefficient. This metric characterizes how edges of a given node are distributed across modules:

8 $${P}_{i}=1-\sum _{m=1}^{M}\,{(\frac{{K}_{i}(m)}{{K}_{i}})}^{2}$$

where M is the number of modules, ${K}_{i}(m)$ is the number of edges between node *i* and nodes in module m. Based on the criteria proposed by ref. 57, a provincial hub having most of its links inside its own module has a *P*_*i*_ value lower than 0.3; while a connector hub has a *P*_*i*_ value greater than 0.3. This criterion was used in our study.

In addition to the evaluation of the difference between brain regions according to their hubness, we were also interested in examining the difference between RSNs. To do so, we associated each brain region in the Desikan-Killiany atlas to its corresponding RSN based on the study described by Shirer *et al*.^93^ in which authors identified fourteen functional networks: anterior salience network, auditory network, basal ganglia network, dorsal default mode network, higher visual network, language network, left executive control network, sensorimotor network, posterior salience network, precunues network, primary visual network, right executive control network, ventral default mode network, and visuospatial network. In our study, we focused on five RSNs: the DMN was obtained by combining the regions of the dorsal and the ventral default mode network, the SAN was obtained by associating all the regions in anterior and posterior salience networks. The combination of the higher and primary visual networks yields to our VIS network.

### Statistical tests

To statistically identify the significant nodes in terms of each graph metric, we quantified the difference between nodes distributions using a Wilcoxon test for continuous data distribution (metrics distribution, transition matrices) and a chi-squared test for binary data distribution (affiliation of connector/non connector, provincial/non provincial hubs). All tests were corrected for multiple comparisons using Bonferroni correction method.

## Electronic supplementary material

Supplementary materials

## References

[1] Fox MD, Raichle ME (2007). Spontaneous fluctuations in brain activity observed with functional magnetic resonance imaging. Nat Rev Neurosci.

[2] Raichle ME (2001). A default mode of brain function. Proceedings of the National Academy of Sciences of the United States of America.

[3] Biswal B, Yetkin FZ, Haughton VM, Hyde JS (1995). Functional connectivity in the motor cortex of resting human brain using echo-planar MRI. Magn. Reson. Med..

[4] Sporns, O. Networks of the Brain: Quantitative Analysis and Modeling. *Notes* (2010).

[5] Biswal BB, Van Kylen J, Hyde JS (1997). Simultaneous assessment of flow and BOLD signals in resting-state functional connectivity maps. NMR Biomed..

[6] Cordes D (2001). Frequencies contributing to functional connectivity in the cerebral cortex in ‘resting-state’ data. Am. J. Neuroradiol..

[7] Cordes D (2000). Mapping functionally related regions of brain with functional connectivity MR imaging. Am. J. Neuroradiol..

[8] Damoiseaux JS (2006). Consistent resting-state networks across healthy subjects. Proc. Natl. Acad. Sci. USA.

[9] De Luca M, Smith S, De Stefano N, Federico A, Matthews PM (2005). Blood oxygenation level dependent contrast resting state networks are relevant to functional activity in the neocortical sensorimotor system. Exp. Brain Res..

[10] Greicius MD, Krasnow B, Reiss AL, Menon V (2003). Functional connectivity in the resting brain: a network analysis of the default mode hypothesis. Proc. Natl. Acad. Sci. USA.

[11] Lowe MJ, Mock BJ, Sorenson JA (1998). Functional Connectivity in Single and Multislice Echoplanar Imaging Using Resting-State Fluctuations. Neuroimage.

[12] Xiong J, Parsons LM, Gao JH, Fox PT (1999). Interregional connectivity to primary motor cortex revealed using MRI resting state images. Hum. Brain Mapp..

[13] van den Heuvel, M., Mandl, R. & Pol, H. H. Normalized cut group clustering of resting-state fMRI data. *PLoS One* **3** (2008). 10.1371/journal.pone.0002001 PMC2291558 18431486

[14] Mantini D, Perrucci MG, Del Gratta C, Romani GL, Corbetta M (2007). Electrophysiological signatures of resting state networks in the human brain. Proc. Natl. Acad. Sci. USA.

[15] Brookes MJ (2011). Measuring functional connectivity using MEG: Methodology and comparison with fcMRI. Neuroimage.

[16] de Pasquale F (2012). A Cortical Core for Dynamic Integration of Functional Networks in the Resting Human Brain. Neuron.

[17] Liu Z, Fukunaga M, de Zwart JA, Duyn JH (2010). Large-scale spontaneous fluctuations and correlations in brain electrical activity observed with magnetoencephalography. Neuroimage.

[18] Gong G (2009). Mapping anatomical connectivity patterns of human cerebral cortex using *in vivo* diffusion tensor imaging tractography. Cereb. Cortex.

[19] Iturria-Medina Y, Sotero RC, Canales-Rodriguez EJ, Aleman-Gumez Y, Melie-Garcia L (2008). Studying the human brain anatomical network via diffusion-weighted MRI and Graph Theory. Neuroimage.

[20] Jahanshad N (2013). Genome-wide scan of healthy human connectome discovers SPON1 gene variant influencing dementia severity. Proc. Natl. Acad. Sci. USA.

[21] Li L (2013). Mapping putative hubs in human, chimpanzee and rhesus macaque connectomes via diffusion tractography. Neuroimage.

[22] Nijhuis, E. H. J., van Cappellen van Walsum, A. M. & Norris, D. G. Topographic Hub Maps of the Human Structural Neocortical Network. *PLoS One* **8** (2013). 10.1371/journal.pone.0065511 PMC3677881 23935801

[23] van den Heuvel MP, Kahn RS, Goñi J, Sporns O (2012). High-cost, high-capacity backbone for global brain communication. Proc. Natl. Acad. Sci. USA.

[24] van den Heuvel MP, Sporns O (2013). An anatomical substrate for integration among functional networks in human cortex. J. Neurosci..

[25] van Horn, J. D. *et al*. Mapping connectivity damage in the case of phineas gage. *PLoS One* **7** (2012). 10.1371/journal.pone.0037454 PMC3353935 22616011

[26] Zalesky A (2010). Whole-brain anatomical networks: Does the choice of nodes matter?. Neuroimage.

[27] Hagmann P (2008). Mapping the structural core of human cerebral cortex. PLoS Biol..

[28] Bola M, Sabel BA (2015). Dynamic reorganization of brain functional networks during cognition. Neuroimage.

[29] Tomasi D, Volkow ND (2011). Association between Functional Connectivity Hubs and Brain Networks. Cereb. Cortex.

[30] Tomasi D, Volkow ND (2010). Functional connectivity density mapping. Proc. Natl. Acad. Sci. USA.

[31] Zuo XN (2012). Network centrality in the human functional connectome. Cereb. Cortex.

[32] Kabbara, A., Falou, W. El Khalil, M., Wendling, F. & Hassan, M. Graph analysis of spontaneous brain network using EEG source connectivity. *arXiv Prepr*. *arXiv1607*.*00952* (2016).

[33] van den Heuvel MP, Sporns O (2011). Rich-Club Organization of the Human Connectome. J. Neurosci..

[34] de Reus Ma, van den Heuvel MP (2013). Rich club organization and intermodule communication in the cat connectome. J. Neurosci..

[35] Collin G, Sporns O, Mandl RCW, van den Heuvel MP (2013). Structural and Functional Aspects Relating to Cost and Benefit of Rich Club Organization in the Human Cerebral Cortex. Cereb. Cortex.

[36] Chang C, Liu Z, Chen MC, Liu X, Duyn JH (2013). EEG correlates of time-varying BOLD functional connectivity. Neuroimage.

[37] Brookes MJ (2014). Measuring temporal, spectral and spatial changes in electrophysiological brain network connectivity. Neuroimage.

[38] Hutchison RM (2013). Dynamic functional connectivity: Promise, issues, and interpretations. Neuroimage.

[39] Allen EA (2014). Tracking whole-brain connectivity dynamics in the resting state. Cereb. Cortex.

[40] Baker, A. P. *et al*. Fast transient networks in spontaneous human brain activity. *Elife* **2014** (2014). 10.7554/eLife.01867 PMC3965210 24668169

[41] de Pasquale F (2010). Temporal dynamics of spontaneous MEG activity in brain networks. Proc. Natl. Acad. Sci. USA.

[42] de Pasquale, F. *et al*.A Dynamic Core Network and Global Efficiency in the Resting Human Brain. *Cereb*. *Cortex* bhv185, doi:10.1093/cercor/bhv185 (2015). 10.1093/cercor/bhv185 PMC5027996 26347485

[43] Hassan M (2015). Dynamic reorganization of functional brain networks during picture naming. Cortex.

[44] Hassan, M., Dufor, O., Merlet, I., Berrou, C. & Wendling, F. EEG source connectivity analysis: From dense array recordings to brain networks. *PLoS One* **9** (2014). 10.1371/journal.pone.0105041 PMC4130623 25115932

[45] Lachaux J-P (2000). Studying single-trials of phase synchronous activity in the brain. Int. J. Bifurc. Chaos.

[46] Achard S (2012). Hubs of brain functional networks are radically reorganized in comatose patients. Proc. Natl. Acad. Sci. USA.

[47] Achard S, Salvador R, Whitcher B, Suckling J, Bullmore E (2006). A resilient, low-frequency, small-world human brain functional network with highly connected association cortical hubs. J. Neurosci..

[48] Alstott, J., Breakspear, M., Hagmann, P., Cammoun, L. & Sporns, O. Modeling the impact of lesions in the human brain. *PLoS Comput*. *Biol*. **5** (2009). 10.1371/journal.pcbi.1000408 PMC2688028 19521503

[49] Cole MW, Pathak S, Schneider W (2010). Identifying the brain’s most globally connected regions. Neuroimage.

[50] Gol’dshtein V, Koganov GA, Surdutovich GI (2004). Vulnerability and Hierarchy of Complex Networks. Physics (College. Park. Md)..

[51] Freeman LC (1977). A Set of Measures of Centrality Based on Betweenness. Sociometry.

[52] Harriger, L., van den Heuvel, M. P. & Sporns, O. Rich Club Organization of Macaque Cerebral Cortex and Its Role in Network Communication. *PLoS One* **7** (2012). 10.1371/journal.pone.0046497 PMC3460908 23029538

[53] He, Y. *et al*. Uncovering intrinsic modular organization of spontaneous brain activity in humans. *PLoS One* **4** (2009). 10.1371/journal.pone.0005226 PMC2668183 19381298

[54] Sporns, O., Honey, C. J. & Kotter, R. Identification and classification of hubs in brain networks. *PLoS One* **2** (2007). 10.1371/journal.pone.0001049 PMC2013941 17940613

[55] Costa LF, Rodrigues FA, Travieso G, Villas Boas PR (2007). Characterization of complex networks: A survey of measurements. Adv. Phys..

[56] Kaiser M, Hilgetag CC (2004). Edge vulnerability in neural and metabolic networks. Biol. Cybern..

[57] Guimerà R, Nunes Amaral LA (2005). Functional cartography of complex metabolic networks. Nature.

[58] Moussa MN (2011). Changes in Cognitive State Alter Human Functional Brain Networks. Front. Hum. Neurosci..

[59] Meunier D, Achard S, Morcom A, Bullmore E (2009). Age-related changes in modular organization of human brain functional networks. Neuroimage.

[60] Power JD, Schlaggar BL, Lessov-Schlaggar CN, Petersen SE (2013). Evidence for hubs in human functional brain networks. Neuron.

[61] Desikan RS (2006). An automated labeling system for subdividing the human cerebral cortex on MRI scans into gyral based regions of interest. Neuroimage.

[62] Hassan, M. *et al*. Identification of Interictal Epileptic Networks from Dense-EEG. *Brain Topography* 1–17, doi:10.1007/s10548-016-0517-z (2016). 10.1007/s10548-016-0517-z 27549639

[63] Buckner RL (2009). Cortical hubs revealed by intrinsic functional connectivity: mapping, assessment of stability, and relation to Alzheimer’s disease. J. Neurosci..

[64] Cole MW, Yarkoni T, Repovš G, Anticevic A, Braver TS (2012). Global Connectivity of Prefrontal Cortex Predicts Cognitive Control and Intelligence. J. Neurosci..

[65] Lohmann, G. *et al*. Eigenvector centrality mapping for analyzing connectivity patterns in fMRI data of the human brain. *PLoS One* **5** (2010). 10.1371/journal.pone.0010232 PMC2860504 20436911

[66] Joyce, K. E., Laurienti, P. J., Burdette, J. H. & Hayasaka, S. A new measure of centrality for brain networks. *PLoS One* **5** (2010). 10.1371/journal.pone.0012200 PMC2922375 20808943

[67] Raichle ME, Snyder AZ (2007). A default mode of brain function: A brief history of an evolving idea. Neuroimage.

[68] Mason MF (2007). Wandering Minds: The Default Network and Stimulus-Independent Thought. Science (80-.)..

[69] Calhoun VD, Miller R, Pearlson G, Adali T (2014). The Chronnectome: Time-Varying Connectivity Networks as the Next Frontier in fMRI Data Discovery. Neuron.

[70] Garrison KA, Scheinost D, Finn ES, Shen X, Constable RT (2015). The (in)stability of functional brain network measures across thresholds. Neuroimage.

[71] Brookes MJ (2011). Investigating the electrophysiological basis of resting state networks using magnetoencephalography. Proc. Natl. Acad. Sci. USA.

[72] Hipp JF, Hawellek DJ, Corbetta M, Siegel M, Engel AK (2012). Large-scale cortical correlation structure of spontaneous oscillatory activity. Nat. Neurosci..

[73] Becker H (2015). Brain-source imaging: From sparse to tensor models. IEEE Signal Process. Mag..

[74] Brookes MJ, Woolrich MW, Barnes GR (2012). Measuring functional connectivity in MEG: A multivariate approach insensitive to linear source leakage. Neuroimage.

[75] Delorme A, Makeig S (2004). EEGLAB: An open source toolbox for analysis of single-trial EEG dynamics including independent component analysis. J. Neurosci. Methods.

[76] Tadel, F., Baillet, S., Mosher, J. C., Pantazis, D. & Leahy, R. M. Brainstorm: A user-friendly application for MEG/EEG analysis. *Comput*. *Intell*. *Neurosci*. **2011** (2011). 10.1155/2011/879716 PMC3090754 21584256

[77] Fischl B (2012). FreeSurfer. NeuroImage.

[78] Gramfort A, Papadopoulo T, Olivi E, Clerc M (2010). OpenMEEG: opensource software for quasistatic bioelectromagnetics. Biomed. Eng. Online.

[79] Hamalainen MS, Ilmoniemi RJ (1994). Interpreting magnetic fields of the brain: minimum norm estimates. Med. Biol. Eng. Comput..

[80] Hassan, M., Shamas, M., Khalil, M., Falou, W. & El Wendling, F. EEGNET: An open source tool for analyzing and visualizing M/EEG connectome. *PLoS One* **10** (2015). 10.1371/journal.pone.0138297 PMC4574940 26379232

[81] Barrat A, Barthélemy M, Pastor-Satorras R, Vespignani A (2004). The architecture of complex weighted networks. Proc. Natl. Acad. Sci. USA.

[82] Watts DJ, Strogatz SH (1998). Collective dynamics of ‘small-world’ networks. Nature.

[83] Rubinov M, Sporns O (2010). Complex network measures of brain connectivity: Uses and interpretations. Neuroimage.

[84] Xia, M., Wang, J. & He, Y. BrainNet Viewer: A Network Visualization Tool for Human Brain Connectomics. *PLoS One* **8** (2013). 10.1371/journal.pone.0068910 PMC3701683 23861951

[85] Good, B. H., De Montjoye, Y. A. & Clauset, A. Performance of modularity maximization in practical contexts. *Phys*. *Rev*. *E* - *Stat*. *Nonlinear*, *Soft Matter Phys*. **81** (2010). 10.1103/PhysRevE.81.046106 20481785

[86] Girvan M, Newman MEJ (2002). Community structure in social and biological networks. Proc. Natl. Acad. Sci. USA.

[87] Blondel VD, Guillaume J-L, Lambiotte R, Lefebvre E (2008). Fast unfolding of communities in large networks. J. Stat. Mech. Theory Exp..

[88] Rosvall M, Axelsson D, Bergstrom CT (2009). The map equation. Eur. Phys. J. Spec. Top..

[89] Lancichinetti A, Fortunato S (2012). Consensus clustering in complex networks. Sci. Rep..

[90] Rubinov M, Sporns O (2011). Weight-conserving characterization of complex functional brain networks. Neuroimage.

[91] Sales-Pardo M, Guimerà R, Moreira AA, Amaral LAN (2007). Correction for Sales-Pardo *et al*., Extracting the hierarchical organization of complex systems. Proc. Natl. Acad. Sci. USA.

[92] Bassett, D. S. *et al*. Robust detection of dynamic community structure in networks. *Chaos* **23** (2013). 10.1063/1.4790830 PMC3618100 23556979

[93] Shirer WR, Ryali S, Rykhlevskaia E, Menon V, Greicius MD (2012). Decoding subject-driven cognitive states with whole-brain connectivity patterns. Cereb. Cortex.

