# Supplementary material for: The dynamic functional core network of the human brain at rest
Source: Sci Rep. 2017 Jun 7;7:2936. doi: 10.1038/s41598-017-03420-6 (PMC5462789; doi:10.1038/s41598-017-03420-6)
Supplement: Supplementary file 1 — Supplementary materials [file 41598_2017_3420_MOESM1_ESM.pdf]

Supplementary materials for

The dynamic functional core network of the human brain at rest

Kabbara A.[1, 2, 3,4,*], EL Falou W.[3,4], Khalil M.[3,4], Wendling F.[1, 2], Hassan M.[1, 2]

[1] INSERM, U1099, F-35000 Rennes, France

[2] University of Rennes 1, LTSI, F-35000 Rennes, France

[3] Azm research center in biotechnology, EDST, Lebanese University, Lebanon

[4] CRSI research center, Faculty of engineering, Lebanese University, Lebanon

[*] Corresponding author: aya.kabbara@etudiant.univ-rennes1.fr

Supplemental Data items

Acronyms	Name	RSN
iCC L	isthmuscingulate L	DMN
iCC R	isthmuscingulate R	DMN
MOF L	medialorbitofrontal L	DMN
MOF R	medialorbitofrontal R	DMN
PCC L	posteriorcingulate L	DMN
PCC R	posteriorcingulate R	DMN
PCUN L	precuneus L	DMN
PCUN R	precuneus R	DMN
rACC L	rostralanteriorcingulate L	DMN
rACC R	rostralanteriorcingulate R	DMN
LOF L	lateralorbitofrontal L	DMN
LOF R	lateralorbitofrontal R	DMN
paraH L	parahippocampal L	DMN
paraH R	parahippocampal R	DMN
cACC L	cAUDalanteriorcingulate L	DAN
cACC R	cAUDalanteriorcingulate R	DMN
ITG L	inferiortemporal L	DAN
ITG R	inferiortemporal R	DAN
MTG L	middletemporal L	DAN
MTG R	middletemporal R	DAN
pOPER L	parsopercularis L	DAN
pOPER R	parsopercularis R	DAN
pORB L	parsorbitalis L	DAN
pORB R	parsorbitalis R	DAN
pTRI L	parstriangularis L	DAN
pTRI R	parstriangularis R	DAN
INS L	insula L	SAN
INS R	insula R	SAN
rMFG L	rostralmiddlefrontal L	SAN
rMFG R	rostralmiddlefrontal R	SAN
SMAR L	supramarginal L	SAN
SMAR R	supramarginal R	SAN
cMFG L	caudalmiddlefrontal L	SAN
cMFG R	caudalmiddlefrontal R	SAN

Acronyms	Name	RSN
STG L	superiortemporal L	AUD
STG R	superiortemporal R	AUD
CUN L	cuneus L	VIS
CUN R	cuneus R	VIS
LOG L	lateraloccipital L	VIS
LOG R	lateraloccipital R	VIS
FUS R	fusiform R	VIS
FUS L	fusiform L	VIS
LING L	lingual L	VIS
LING R	lingual R	VIS
BSTS L	bankssts L	other
BSTS R	bankssts R	other
ENT L	entorhinal L	other
ENT R	entorhinal R	other
FP L	frontalpole L	other
FP R	frontalpole R	other
IPL L	inferiorparietal L	other
IPL R	inferiorparietal R	other
sFG L	superiorfrontal L	other
sFG R	superiorfrontal R	other
paraC L	paracentral L	other
paraC R	paracentral R	other
periCAL L	pericalcarine L	other
periCAL R	pericalcarine R	other
postC L	postcentral L	other
postC R	postcentral R	other
preC L	precentral L	other
preC R	precentral R	other
SPL L	superiorparietal L	other
SPL R	superiorparietal R	other
TP L	temporalpole L	other
TP R	temporalpole R	other
TT L	transversetemporal L	other
TT R	transversetemporal R	other

Table S1. Anatomic regions-of-interest (ROIs) included in the analysis, as derived from the Desikan Killiany atlas, and their affiliation to RSNs.

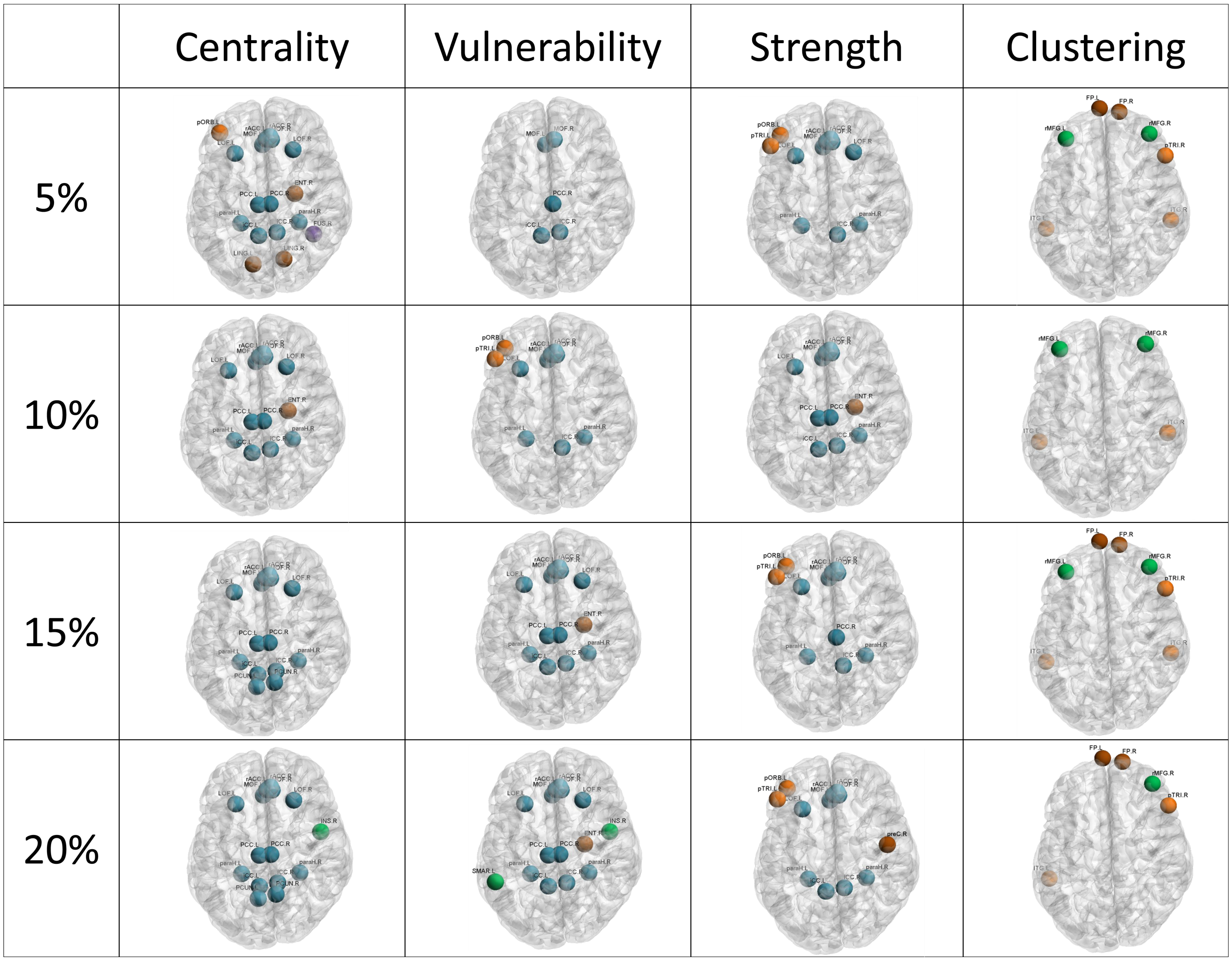

Figure S1. The locations of the significant brain regions on the cortical surface for the different thresholds (5%, 10%, 15%, 20%) in terms of centrality, vulnerability, strength and clustering coefficient. The color of the node corresponds to which RSN the region is assigned. Names and abbreviations of the brain regions are listed in table S1.

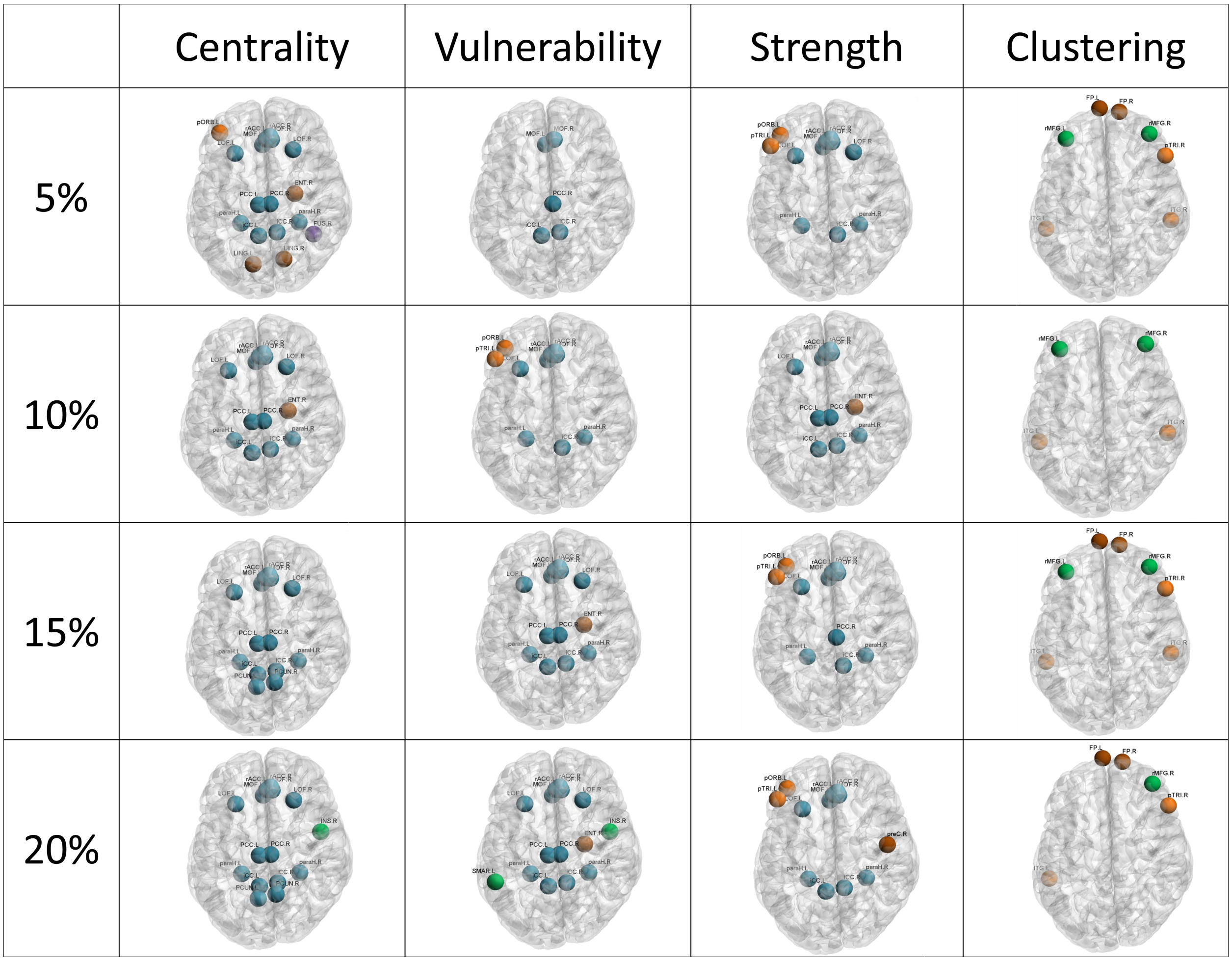

Figure S2. The locations of the significant brain regions on the cortical surface for different window size (300ms, 1s, 2s, 10s) in terms of centrality, vulnerability, strength and clustering coefficient. The color of the node corresponds to which RSN the region is assigned. Names and abbreviations of the brain regions are listed in table S1.

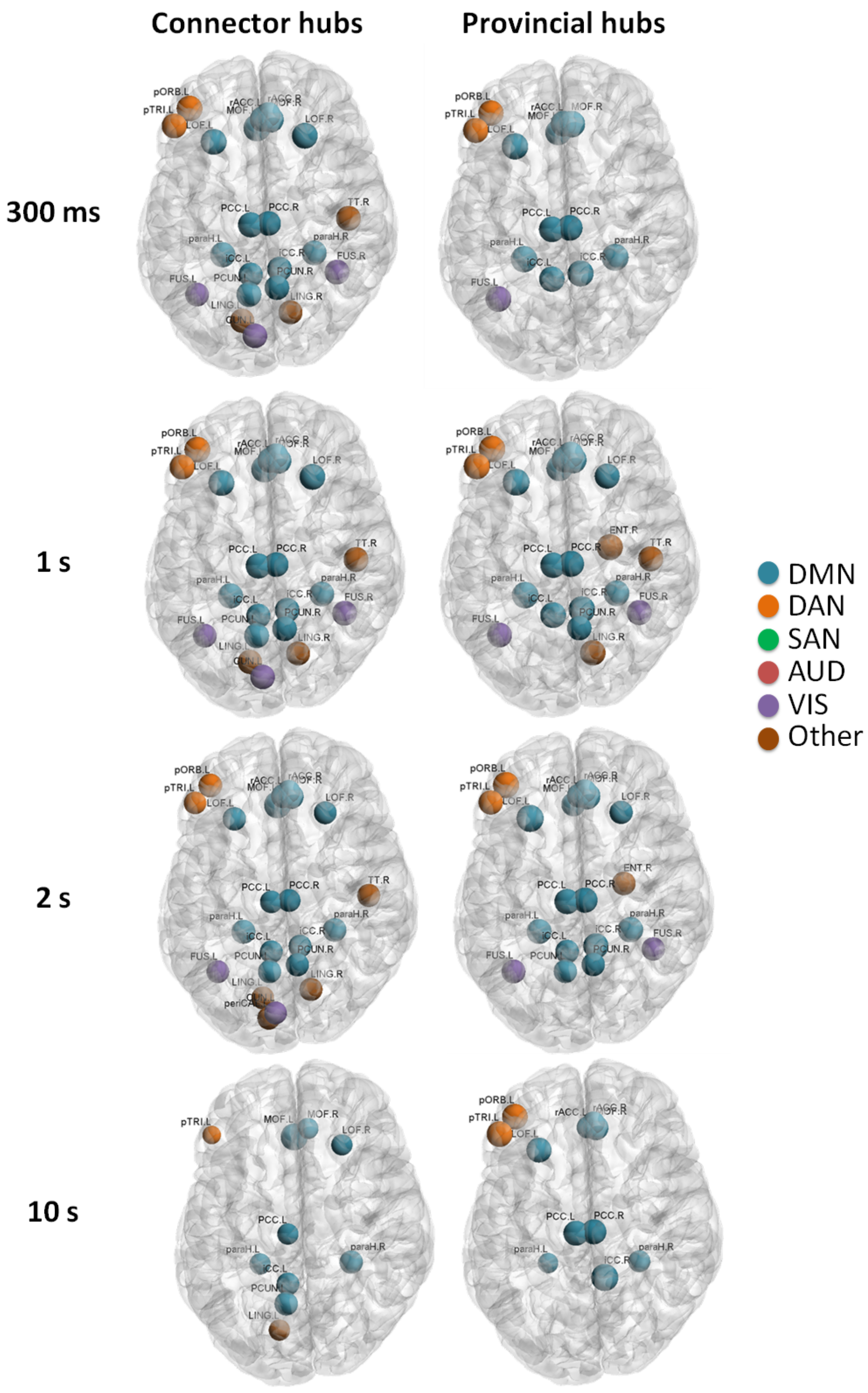

Figure S3. The spatial distributions of significant provincial hubs, and significant connector hubs for different window size. The color of the node corresponds to which RSN this node is assigned.

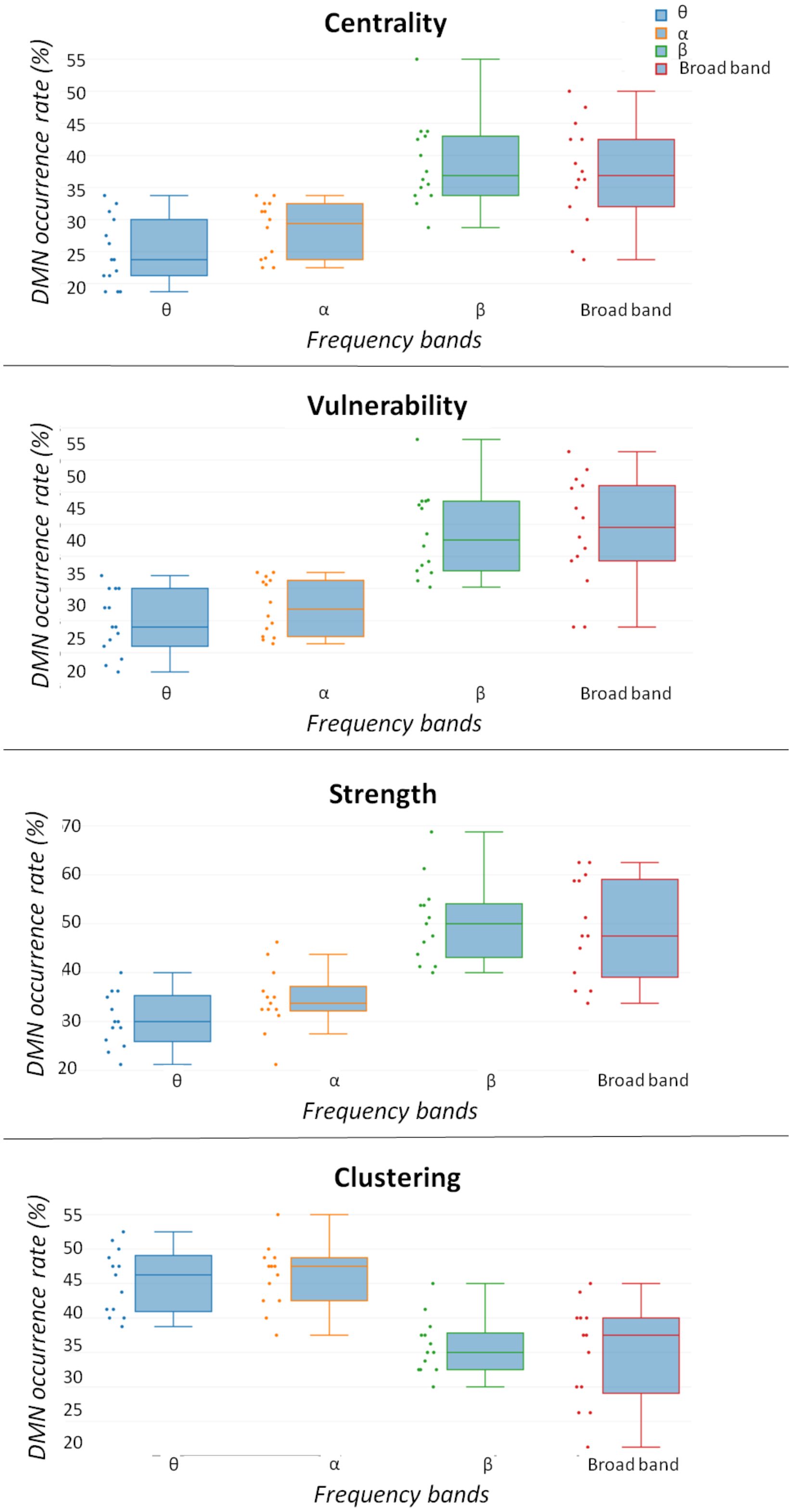

Figure S4. The fractional occupancy of DMN in each of Θ (3-7 Hz), α (7-13 Hz), β (14-25 Hz) and broad-band (3-45 Hz) in term of centrality, strength, vulnerability and clustering.
